# Identification and Candidate Gene Analysis of a Novel Phytophthora Resistance Gene *Rps10* in a Chinese Soybean Cultivar

**DOI:** 10.1371/journal.pone.0069799

**Published:** 2013-07-25

**Authors:** Jiqing Zhang, Changjian Xia, Canxing Duan, Suli Sun, Xiaoming Wang, Xiaofei Wu, Zhendong Zhu

**Affiliations:** MOA Key Lab of Soybean Biology (Beijing), the National Key Facility for Crop Gene Resources and Genetic Improvement, Institute of Crop Science, Chinese Academy of Agricultural Sciences, Beijing, People's Republic of China; Nanjing Agricultural University, China

## Abstract

Resistance to *Phytophthora sojae* isolate PsMC1 was evaluated in 102 F_2∶3_ families derived from a cross between the resistant soybean cultivar Wandou 15 and the susceptible cultivar Williams and genotyped using simple sequence repeat (SSR) markers. The segregation ratio of resistant, segregating, and susceptible phenotypes in the population suggested that the resistance in Wandou 15 was dominant and monogenic. Twenty-six polymorphic SSR markers were identified on soybean chromosome 17 (Molecular linkage group D2; MLG D2), which were linked to the resistance gene based on bulked segregation analysis (BSA). Markers Sattwd15-24/25 and Sattwd15-47 flanked the resistance gene at a distance of 0.5 cM and 0.8 cM, respectively. Two cosegregating markers, Sattwd15-28 and Sattwd15-32, were also screened in this region. This is the first *Rps* resistance gene mapped on chromosome 17, which is designated as *Rps10*. Eight putative genes were found in the mapped region between markers Sattwd15-24/25 and Sattwd15-47. Among them, two candidate genes encoding serine/threonine (Ser/Thr) protein kinases in Wandou 15 and Williams were identified and sequenced. And the differences in genomic sequence and the putative amino acid sequence, respectively, were identified within each candidate gene between Wandou 15 and Williams. This novel gene *Rps10* and the linked markers should be useful in developing soybean cultivars with durable resistance to *P*. *sojae*.

## Introduction

Phytophthora root rot (PRR), caused by the *Phytophthora sojae* Kaufmann & Gerdemann, is one of the most destructive diseases of soybean (*Glycine max* (L.) Merr.) [1]. Since it was first reported in the United States in 1948, PRR has been observed in other soybean-producing areas, including Australia, Europe, Asia, and Africa [1], [2]. In China, PRR was first recorded in 1991 in Heilongjiang Province [3], and since then, this disease has occurred in almost all soybean production-areas of the province [4], [5]. *P*. *sojae* can infect soybean plants at any stage of growth. It not only kills seedlings and young plants, it also causes wilting and death of adult plants in later stages [6]. PRR reduces soybean yield by 10–40%, and severe infection can result in a complete yield loss during severe epiphytotic outbreaks [7], [8].

The most effective method to control PRR is to plant resistant cultivars, and genes conferring resistance to *P*. *sojae* (*Rps*) have been widely used in commercial soybean cultivars [1], [9]. To date, twenty *Rps* genes/alleles, involved 14 loci distributed on six different soybean chromosomes, have been reported, including *Rps1*a, *Rps1*b, *Rps1*c, *Rps1*d, *Rps1*k, *Rps2*, *Rps3*a, *Rps3*b, *Rps3*c, *Rps4*, *Rps5*, *Rps6*, *Rps7*, *Rps8*, *Rps9*, *RpsYB30*, *RpsYD25*, *RpsZS18*, *RpsSN10*, and the *Rps* gene in Waseshiroge [10]–[16]. Following deployment of each single *Rps* gene, races of *P*. *sojae* were subsequently identified that were virulent to plants carrying the *Rps* gene. Single *Rps* genes have been effective for 8 to 15 years, depending on inoculum density and environmental conditions [17]. Chen et al. [18] and Zhu et al. [19] reported that most of the *Rps* genes (except *Rps1*c and *Rps1*k) were not effective against the Chinese populations of *P*. *sojae*.

*P*. *sojae* interacts with soybean in a gene-for-gene model, and 55 physiological races of *P*. *sojae*, and many more pathotypes, have been identified in the United States and other countries [20]–[22]. The populations of *P*. *sojae* in China have highly diverse virulence, as in the United States [4], [5], [19]. Zhang et al. [5] found 12 races of *P*. *sojae* present in the Heilongjiang Province of China and identified 14 intermediate reaction types on a set of eight soybean differentials. Cui et al. [4] identified 30 virulence pathotypes from the isolates of Heilongjiang Province that were not characterized to any race based on the published race definitions. As the complexity of the virulence pathotypes of *P*. *sojae* continues to increase in soybean fields, identification and incorporation of new sources with stable and durable resistance into commercial cultivars using marker-assisted selection (MAS) in soybean breeding programs will be essential to control this disease effectively.

The soybean genome has been sequenced and the reference sequence could be used for developing new markers and identifying candidate genes [23], [24]. Using the soybean genome sequence, Sugimoto et al. [24] localized an *Rps* gene to a 2.5 cM region of chromosome 3. Saghai Maroof et al. [23] mapped *Rsv4* to a physical interval of less than 100 kb on chromosome 2. Sequence analysis revealed that this region contained several predicted transcription factors and unknown protein products, indicating that *Rsv4* likely belonged to a new class of resistance genes that interfere with viral infection and cell-to-cell movement, and delay vascular movement, which differed from the *Rsv1* and *Rsv3* genes, which were characterized as nucleotide binding site-leucine rich repeat (NBS-LRR) type proteins.

Soybean cultivar Wandou 15 was registered in Anhui province in 1996 and is fatty, rich in protein, matures early and is resistant to soybean mosaic virus and downy mildew; therefore, it has been widely planted in Huaihe and the middle-lower Yangtze areas in China [25]. Zhu et al. [26] reported that Wandou 15 was resistant to two new races of *P*. *sojae*. Chen et al. [18] postulated that the cultivar carried gene combinations of *Rps1*c or *Rps1*k and *Rps4*. Recently, Xia et al. [27] suggested that Wandou 15 might carry a novel *Rps* gene.

Here, we report (1) the inheritance of Phytophthora resistance in Wandou 15 by investigating phenotypic data; (2) the fine mapping of the *Rps* gene with new SSR markers in the genomic regions associated with PRR resistance; and (3) the prediction and cloning of the candidate *Rps* gene(s) for PRR resistance in Wandou 15.

## Results

### Phenotype analysis for mapping population

**Figure 1:**
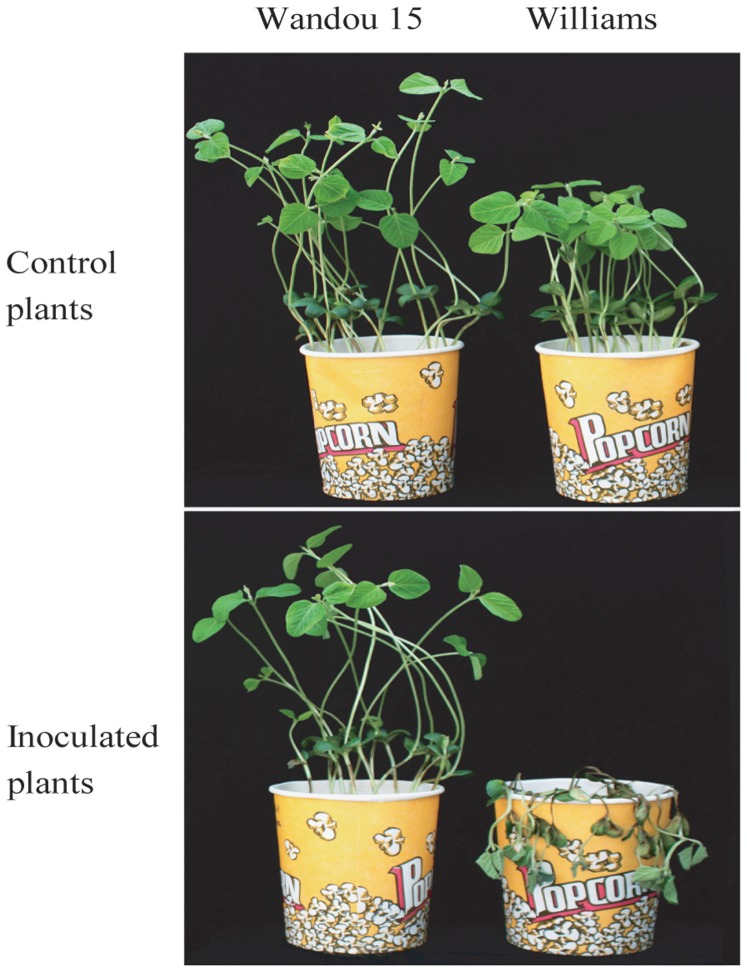
Phytophthora resistance phenotype of the soybean cultivars Wandou 15 and Williams were tested with *Phytophthora sojae* isolate PsMC1 using the hypocotyl-inoculation technique.

The resistant parent Wandou 15 plants showed no symptoms in response to isolate PsMC1 at 6 days post inoculation (DPI), while the susceptible parent Williams plants showed severe rot at the inoculated location at 6 DPI and all plants ultimately died (Figure 1). Among the 102 F_2∶3_ families of the mapping population, 31 families were identified as homozygous-resistant (R), 29 families were homozygous-susceptible (S) and 42 families were segregating (Rs) for isolate PsMC1, according to mortality (Table 1). A segregation ratio of 31∶42∶29 in the F_2∶3_ population fitted well with the genetic model of 1∶2∶1 ratio (χ^2^ = 3.25, *p* = 0.20), indicating that Phytophthora resistance in Wandou 15 is controlled by a single dominant locus.

**Table 1:** Genetic segregation in response to isolate PsMC1 in an F_2∶3_ population derived from a cross between Wandou 15 and Williams.

Cultivar and the cross	Generation	Amount	Observed number			Expected ratio and Goodness of fit		
			R	Rs	S	(R∶Rs∶S)	?^2^	*P*
Wandou 15	P_1_	20	20	-	-			
Williams	P_2_	20	-	-	20			
Williams×Wandou 15	F_2∶3_	102	31	42	29	1∶2∶1	3.25	0.20

### Genetic mapping of the *Rps* gene in Wandou 15

**Figure 2:**
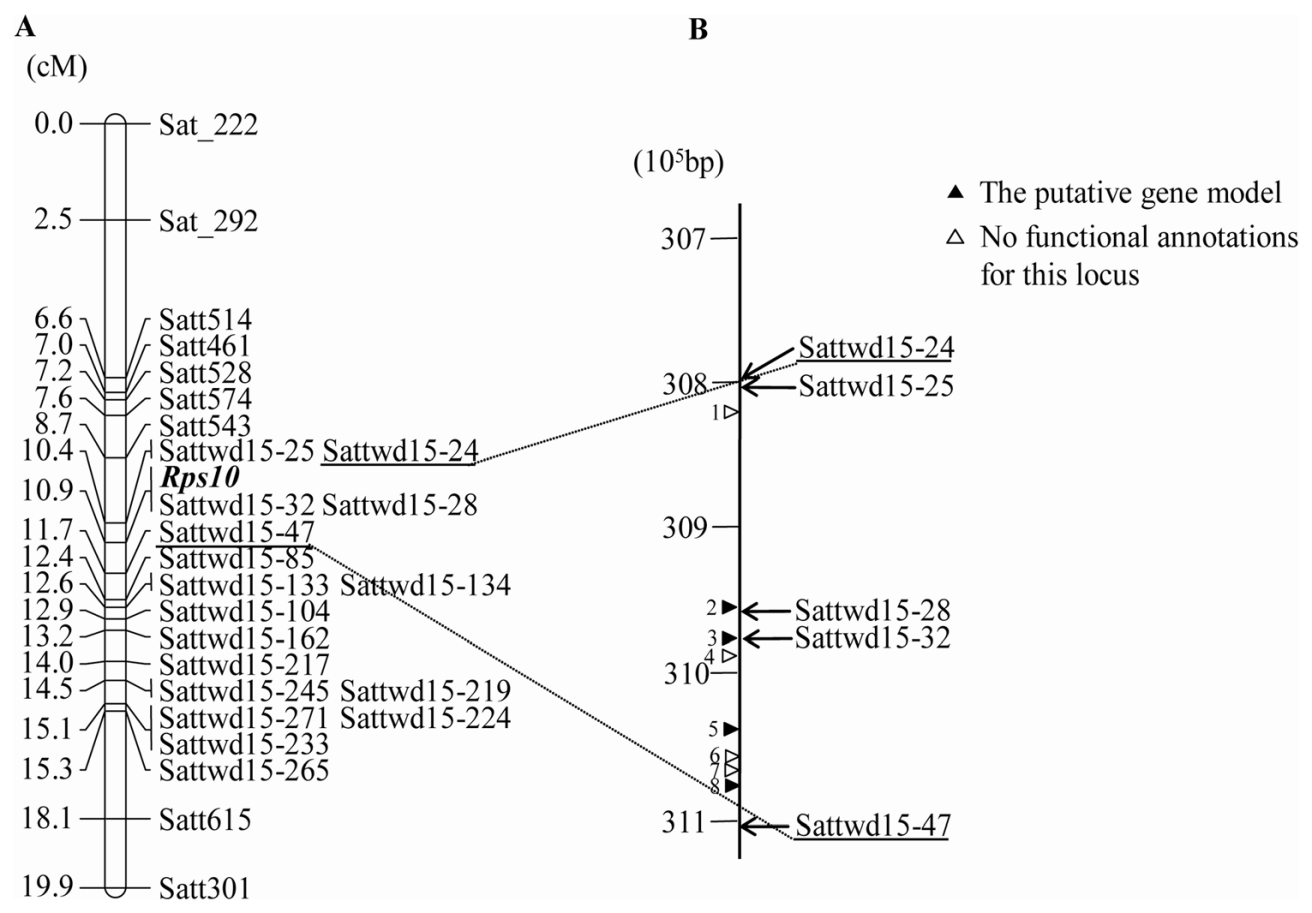
Linkage map of the region surrounding the Phytophthora resistance gene *Rps10* on chromosome 17 (MLG D2) and the gene annotation of the region between the tightly linked markers Sattwd15-24/25 and Sattwd15-47. (A) Genetic map of the Phytophthora resistance gene *Rps10* was deduced from the segregation analysis of 102 F_2∶3_ families derived from the cross of Wandou 15 and Williams. The map was generated in Joinmap v. 4.0 using Kosambi's mapping function (Marker names and distances are on the both side of Linkage map). (B) Physical map and gene annotations between Sattwd15-24/25 and Sattwd15-47 on chromosome 17 of Williams 82. Positions of the tightly linked and cosegregating markers are indicated on the right of the chromosome line, and locations of the putative gene models (filled triangles) and genes with no functional annotation for the locus (open triangles) are indicated on the left of the line.

To map the *Rps* gene in Wandou 15, the SSR markers described in Soybase (http://soybase.org) was screened using the bulk segregation analysis (BSA). Nine SSR markers (Sat_222, Sat_292, Satt514, Satt461, Satt528, Satt574, Satt543, Satt615, and Satt301) on chromosome 17 were screened and showed polymorphisms between Wandou 15 and Williams, as well as the resistant bulk and the susceptible bulk. The linkage analysis further revealed that the *Rps* gene in Wandou 15 was linked to these nine SSR markers and was located between Satt543 and Satt615 at a genetic distance of 8.2 cM (Figure 2A). This was a novel Phytophthora resistance locus that differed from the 14 previously reported *Rps* loci. It was designated as *Rps10*.

To further fine map *Rps10* on chromosome 17, new SSR markers in this region were designed and SSR hunter was used to detect SSRs. BLAST analysis of SSR markers Satt543 and Satt615 detected a 4.29 Mb fragment in the *Glycine max* v1.0 release of the soybean genome sequence in this region. Four hundred and fourteen out of 671 detected SSR loci with more than 10 bp repeat motifs were selected to design primers to survey polymorphisms between the parental cultivars. Among them, 17 new SSR markers showed polymorphisms between Wandou 15 and Williams, and were used to analyze the F_2∶3_ population (Table 2).

**Table 2:** Primers for SSR markers linked to *Rps10*, designed based on the soybean genome sequence from the Phytozome database.

Primer	Forward sequence (5′-3′)	Reverse sequence (5′-3′)	Tm (°C)	Product size (bp)	Repeat motif	Position	Recombination frequency
Sattwd15-24	CTTTGTCCCCTCCTTTAG	TTCAACAAGAAAAGGTAA	50	412	(AT)_17_	30,796,875–30,797,266	0.67
Sattwd15-25	TCATCCAACAACACGCCATT	CTCCATAGTTTGCTTTTA	48	199	(AT)_18_	30,807,628–30,807,827	0.67
Sattwd15-28	GCTTCCTATCACTCTTTGCTG	TTAGGCTAATGATGCTG	48	123	(AT)_23_	30,964,476–30,964,598	0.67
Sattwd15-32	ATCCCTTATTCCCTTCAT	CATAGACCTCCTTCCAAA	47	149	(AAC)_6_	30,983,669–30,983,817	0.67
Sattwd15-47	GAACCTAAACCCACCCAA	TGCTAAAAGGGTGGGAAT	51	128	(TA)_10_	31,107,789–31,107,916	0.67
Sattwd15-85	ATTCAATCCCTTGTCGTT	AAAACGAAGGGCAACC	53	117	(CT)_10_	31,441,547–31,441,653	0.67
Sattwd15-104	ATTCCCTACCCCTTTTGT	GTTTACCGACTTGTTTAT	53	120	(AT)_22_	31,589,038–31,589,157	0.66
Sattwd15-133	AACAACATTCTCCACCAC	ATAAAGTCTTCTCCGCTA	49	184	(CAT)_11_	31,875,642–31,875,825	0.65
Sattwd15-134	CGTAAAAGCGACAGTAAG	CGTTATCTGCTTTATGCTTTTA	51	525	(AT)_21_	31,878,579–31,879,103	0.66
Sattwd15-162	AATCCACCTCCTTCTCAT	TGACGATGATGTAACTAAA	51	134	(TCA)_10_	32,115,297–32,115,430	0.66
Sattwd15-217	GCCAAAACTAAATGCTGA	AGTATGACTTCCATCTTT	49	496	(AT)_11_	32,554,741–32,555,236	0.65
Sattwd15-219	CAATGCCTTCATAGTTTT	TAACATCACTCGTTTCTA	47	218	(AT)_24_	32,577,517–32,577,735	0.65
Sattwd15-224	AAAAGGATGATAAAGTGGAT	TACGATACTCGGTCTTAC	48	217	(AT)_10_	32,627,080–32,627,298	0.65
Sattwd15-233	GTAACGAAGAACCCAAAC	CTTGTGCCTTTGCTCTGC	52	238	(CT)_14_(AT)_17_	32,695,932–32,696,169	0.63
Sattwd15-245	TTGACCAAATGGCAGCAC	GGGATGGAAAATCATAGAA	50	222	(CA)_11_	32,841,971–32,842,192	0.63
Sattwd15-261	GGCAGTTAGTCCTTGTCA	ACTCTTCCAATGGTTTTCT	50	336	(GAA)_5_	33,110,188–33,110,523	0.65
Sattwd15-265	AACTTTCTTGTAACCCTT	TGGTGTTTCAAAAGGGAT	49	237	(AT)_21_	33,204,538–33,204,773	0.60
Sattwd15-271	ATGGTTCTTCTTGGTATT	AGTTCTCCTAACAGTGGG	48	360	(AT)_33_	33,265,825–33,266,184	0.63

Based on the genotype data of the 26 polymorphic SSR markers, *Rps10* was further localized between markers Sattwd15-24/25 and Sattwd15-47 with a genetic distance of 1.3 cM (Figure 2A). Furthermore, two markers, Sattwd15-28 and Sattwd15-32, cosegregated with *Rps10* in this population (Figure 2A).

### Candidate gene prediction and analysis

**Figure 3:**
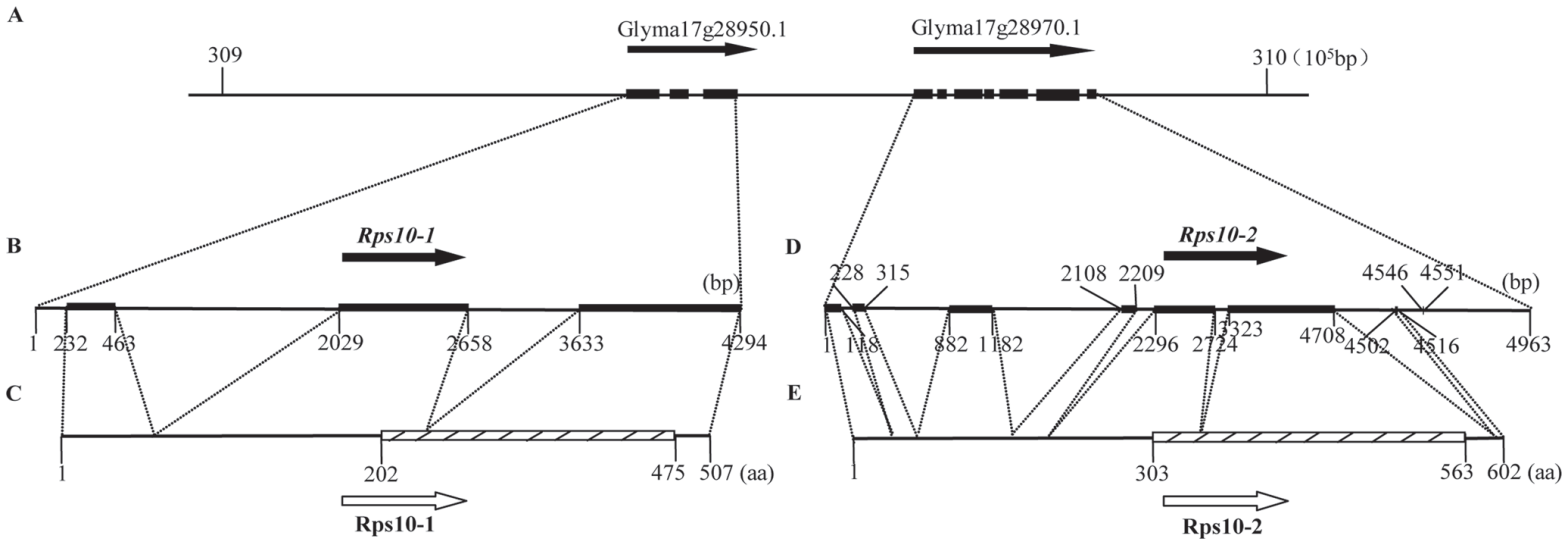
Structures of the gene models Glyma17g28950.1 and Glyma17g28970.1, the candidate genes *Rps10-1* and *Rps10-2*, and the putative proteins Rps10-1 and Rps10-2. (A) The structures of the gene models Glyma17g28950.1, Glyma17g28970.1 from 309×10^5^ to 310×10^5^ bp on chromosome 17 of Williams 82. (B) Predicated structure of the candidate gene *Rps10-1*. (C) Predicted structure of the putative protein Rps10-1. (D) Predicated structure of the candidate gene *Rps10-2*. (E) Predicted structure of the putative protein Rps10-2. Filled rectangular arrows with orientations (from 5′ to 3′) indicate the predicated genes. Filled rectangles indicate the exons. Open rectangular arrows with orientations (N-C) indicate the predicated proteins. The filled rectangle indicates the Ser/Thr protein kinase domain.

To locate the *Rps10* region in the soybean genome sequence, BLASTN searches for the sequences of SSR markers tightly linked to *Rps10* were performed initially against the soybean genome sequence, release *Glycine max* v1.0 (http://www.Phytozome.net). Based on the public soybean physical map [28], the mapped length of the region flanked by markers Sattwd15-24/25 and Sattwd15-47 on chromosome 17 is approximately 311 kb. By inspection of the soybean gene annotation database, eight candidate genes were detected in the mapped region (Figure 2B; Table S1). Four out of the eight genes were annotated with known protein function: one for Prokaryotic DNA topoisomerase, one for Glycosyl transferase family 2, and the other two genes, Glyma17g28950.1 and Glyma17g28970.1, were annotated as serine/threonine (Ser/Thr) protein kinases with plant-type (Figure 2B; Table S1). Furthermore, the two SSR markers Sattwd15-28 and Sattwd15-32 cosegregated with *Rps10* were found in Glyma17g28950.1 and Glyma17g28970.1, respectively. The sequences of Wandou 15 and Williams amplified by both markers shared 92.40–96.64% and 96.05–100% identity with Glyma17g28950.1 and Glyma17g28970.1 genes, respectively (Figure S1A, S1B). Thus, Glyma17g28950.1 and Glyma17g28970.1 were also co-segregated with *Rps10* phenotype, and most likely the referenced candidate genes of *Rps10* (Figure 3A).

### Candidate gene analysis with allelic sequence comparison

To obtain the fragment of genomic DNA of the two candidate genes in Wandou 15, two pairs of primers were designed according to the regions of the 5′-UTR and 3′-UTR of the gene models Glyma17g28950.1 and Glyma17g28970.1, respectively. These two candidate genes of *Rps10* in Wandou 15 were named as *Rps10-1* and *Rps10-2*. The full lengths of *Rps10-1* (GenBank No. JX682937) and *Rps10-2* (GenBank No. JX682938) were 4294 bp and 4963 bp, respectively (Figure 3B, 3D). Compared with the known genomic sequence, *Rps10-1* and *Rps10-2* shared 98.72% and 99.62% identity with the sequences of Glyma17g28950.1 and Glyma17g28970.1 in Williams 82. *Rps10-1* was predicted to have three exons and two introns (Figure 3B), and encoded a putative 507-aa protein (Figure 3C). *Rps10-2* was predicted to have seven exons and six introns (Figure 3D), and encoded a putative 602-aa protein (Figure 3E).

**Figure 4:**
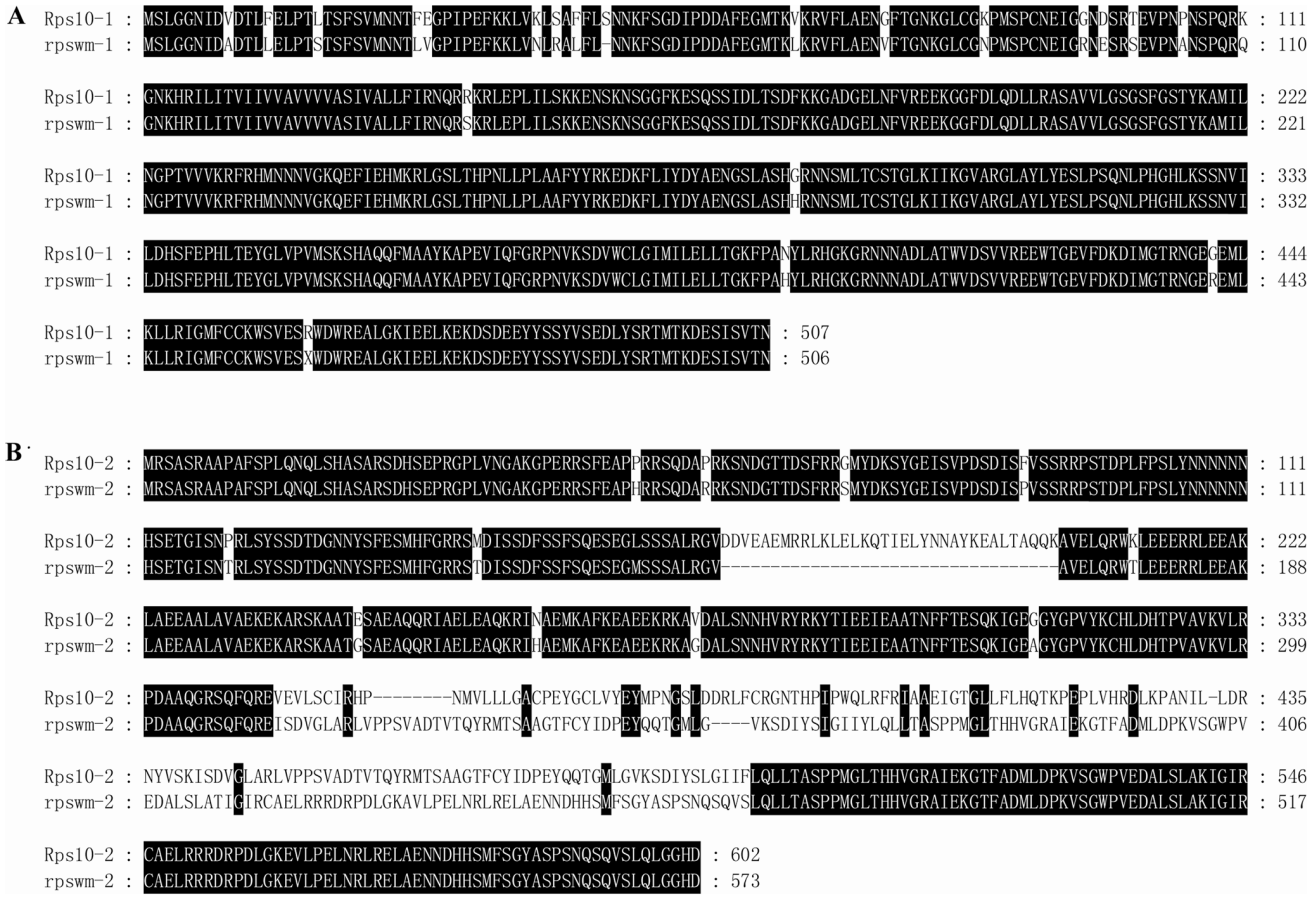
Alignment of the amino acid sequences of the predicted proteins encoded by *Rps10-1*, *Rps10-2*, *rpswm-1* and *rpswm-2* genes. (A) The alignment of amino acid sequences of the predicted proteins encoded by *Rps10-1* and *rpswm-1* genes. (B) The alignment of amino acid sequences of the predicted proteins encoded by *Rps10-2* and *rpswm-2* genes. The black boxes indicate the conserved domains within Rps10-1 and rpswm-1, Rps10-2 and rpswm-2. Open boxes show the amino acids that differ between Rps10-1 and rpswm-1 and Rps10-2 and rpswm-2.

To analyze the susceptible alleles, these two genes were also completely sequenced and investigated for the presence of the characteristic motifs. The allelic sequences *rpswm-1* and *rpswm-2* in Williams were 4272 bp, 4966 bp in length, and shared 98.35% and 98.23% identity with *Rps10-1* and *Rps10-2*, respectively. The *rpswm-1* was predicted to contain three exons and four introns, and was nearly identical in size and sequence with *Rps10*-*1* (Figure S2A). And *rpswm-2* was predicted to contain five exons and six introns, which was significantly different from the structure of the *Rps10-2* (Figure S2B). Comparison of the predicted protein sequences revealed the polymorphisms in the amino acid sequence present between Rps10-1 and rpswm-1, and between Rps10-2 and rpswm-2 (Figure 4). These results indicated that the sequence variations either between Rps10-1 and rpswm-1, or between Rps10-2 and rpswm-2, may account for the resistance to PRR in Wandou 15, or for susceptibility to PRR in Williams.

## Discussion

The results of the present study indicated that resistance to *P*. *sojae* in Wandou 15 is controlled by a single dominant locus, *Rps10*, located on soybean chromosome 17. Thus, *Rps10* is a novel *Rps* gene given that it is the first Phytophthora resistance gene identified on chromosome 17. Two markers Sattwd15-28 and Sattwd15-32 were identified that cosegregated with *Rps10*. Markers Sattwd15-24/25 and Sattwd15-47 were found to flank the gene at a distance of 0.5 and 0.8 cM, respectively. It was theoretically estimated the MAS accuracies would be 100% and 99.2% using both cosegregating markers and flanking markers, respectively.

Several other disease-resistant quantitative trait loci (QTLs) or recessive genes have been mapped at chromosome 17. To the best of our knowledge, three QTLs conferring sudden death syndrome, three QTLs for resistance to soybean cyst nematode, seven QTLs conferring sclerotinia stem rot resistance, *rxp* gene and several QTLs conferring bacterial leaf pustule resistance are also located at different regions of chromosome 17 [29]–[33]. *Rps10* is the first single dominant disease resistance gene identified on chromosome 17 that confers resistance to *P*. *sojae*. Kang et al. [34] reported that only nine NBS-LRR genes were located on this chromosome. These data indicate that *Rps10* was located in a disease resistance gene-poor region.

To date, 19 *Rps* genes/alleles have been reported, but only three *Rps* genes, *Rps1*k, *Rps2* and *Rps4*, have been cloned and characterized as NBS-LRR genes, which involved recognizing the presence of pathogens and ultimately confer resistance [35]–[37]. In this study, eight putative genes were found in the mapped region and the four un-annotated genes out of the eight genes were analyzed by BLAST searching the nucleotide sequence at the NCBI and the putative protein sequence at the EMBL-EBI databases. Glyma17g28740.1 might function as a DNA/RNA polymerase or in nucleic acid binding. Glyma17g28980.1 shared 100% identity with a gene (GenBank No. XM_003551110) encoding the predicted *Glycine max* DNA topoisomerase 1-like. Glyma17g29050.1 might encode the 60S ribosomal protein L30-like predicted by the gene (GenBank No. XM_003552594). However, Glyma17g29040.1 only shared 82% identity in nucleotide sequence with *Glycine max* clone GM_WBc0124O13 (GenBank No. AC236224). There was no similar level of correspondence among the proteins in the EMBL-EBI database. We suggested that Glyma17g28950.1 and Glyma17g28970.1, encoding plant-type Ser/Thr protein kinases, were the candidate genes of *Rps10*. The two candidate genes, *Rps10*-*1* and *Rps10*-*2*, about 14 kb apart, were identified at this region. Using the InterProScan program analysis, their predicted domain structure was: 1) a hydrophobic N-terminal putative signal peptide, and 2) a C-terminal Ser/Thr protein kinase catalytic domain. Additionally, Rps10-1 contained a putative transmembrane domain followed by a membrane transfer-stop signal. These analyses indicated that Rps10-1 and Rps10-2 might be the Ser/Thr protein kinases. The difference in structures between the Ser/Thr genes representing *Rps10* and the NBS-LRR genes *Rps1*k, *Rps2*, and *Rps4* indicated that the mechanism of resistance in the soybean-*P*. *sojae* interaction was likely to be different.

Some resistance genes belonging to the RLK families have been characterized as an intracellular Ser/Thr protein kinase, such as *Pto*, *Xa21*, *Xa26*, *ZmPto* and *NgRLK1* [38]–[42]. Based on the structure of the putative extracellular domain, the plant RLKs were divided into seven classes: the S-locus glycoprotein like (SLG-like), Leucine-Rich Repeats (LRRs), epidermal growth factor-like (EGF-like) repeats, putative carbohydrate-binding lectin (Lectin-like), the tumor necrosis factor receptor (TNFR), pathogenesis-related (PR5) and N-glycosylation site type of RLKs [43]. Interestingly, the N-terminal 116 aa of predicted Rps10-1, and 302 aa of predicted Rps10-2 have no similarities to the extracellular regions of the known RLKs. The kinase domain of Rps10-1 and Rps10-2 showed 22–41% sequence similarity to other plant RLKs (data not shown). In addition, Rps10-1 and Rps10-2 showed a low level of sequence identity (4–5%) with the calcium-dependent protein kinase SK5-like proteins in soybean (data not shown). The above analyses suggested that *Rps10-1* and *Rps10-2* might encode a novel type of plant RLK.

RLKs are unusual membrane-associated plant protein kinases, some of which have important roles in pathogen resistance. For example, the Ser/Thr protein kinase Pto, which confers resistance to *Pseudomonas syringae* pv. *tomato*, is autophosphorylated at the Thr38 and Ser198 sites after monitoring the signal of the interaction between effector AvrPto and tomato Prf. Prf is further phosphorylated by another Ser/Thr kinase Pti1 to induce a series of mitogen-activated protein kinase cascades, resulting in a hypersensitive response (HR) [44]–[47]. The rice Xa21 kinase domain interacts with the protein XB3 via its ankyrin repeat domain and trans-phosphorylation occurs. The physical interaction with XB3 is thought to stabilize the Xa21 protein, thereby maintaining R protein levels and hence the capacity to fully activate the defense response.

The differences in the predicted amino acid sequences between Rps10-1 and rpswm-1, Rps10-2 and rpswm-2 might explain the resistance/susceptibility of the parental cultivars. However, the resistance mechanism of *Rps10* is still ambiguous. The previous revealed that phosphorylation played an important role in regulating components of signaling pathways involved in the HR. Analysis in the KinasePhos database revealed that seven out 44 Ser-phosphorylation sites, and one out 22 Thr- phosphorylation sites, were detected in Rps10-1.Seven out 43 Ser-phosphorylation sites, and one out 15 Thr-phosphorylation sites, were detected in the Rps10-2 (data not shown). These predicted phosphorylation sites are very important for further investigation of the resistance mechanism of *Rps10*. The functional significance of the proposed novel Phytophthora resistance mechanism remains to be elucidated.

## Materials and Methods

### Plant materials

Wandou 15 is a PRR-resistant soybean cultivar, and Williams is a susceptible cultivar. The F_1_ plants from the cross between Wandou 15 and Williams were self-pollinated to produce an F_2_ population. Each F_2_ plant was threshed individually to yield seeds of 102 F_2∶3_ families. The 102 F_2∶3_ families were used to investigate both phenotypic and genetic data to map the resistance gene(s) for PRR.

### *Phytophthora sojae* inoculation and PRR evaluation

The *P*. *sojae* isolate PsMC1 (pathotype 1a, 1c, 1k, 2, 3b, 3c, 4, 5, 6, 7, 8, ZS18) was used as the inoculum to evaluate the phenotype of parental materials and the F_2∶3_ families. Fourteen-day-old seedlings of Wandou 15 (20 individuals), Williams (20 individuals), and each F_2∶3_ family (30 individual seedlings) were inoculated with PsMC1 using the hypocotyl-inoculation technique as described by Haas and Buzzell [48]. The negative control for both parental cultivars was performed in the same way with pure carrot agar slurry instead of PsMC1 inoculum slurry. After inoculation, the plants were placed in a mist room with relative humidity 100% and an average temperature of 25°C for 2 d. Then they were moved to a greenhouse with average temperature 25°C.

The reactions were recorded by the mortality of inoculated plants in each F_2∶3_ family and parental cultivars 6 DPI. A family with 0–20% seedling death was scored as homozygous-resistant (R), a family with 80–100% seedling death was considered homozygous-susceptible (S), while a family with 21–79% was scored as segregating (Rs) [49].

### DNA preparation and pooling for bulk segregation analysis

Equivalent amounts of leaf tissues from 30 seedlings of each family were bulked and placed in liquid nitrogen, and ground into a powder. Genomics DNA was extracted by the Cetyl trimethylammonium bromide method [50]. Resistant and susceptible bulks of DNA for the BSA [51] were prepared by pooling an equal amount of DNA (1 µg) from each of 10 selected homozygous-resistant and 10 susceptible F_2∶3_ families, respectively. The finial concentration of the DNA bulks was adjusted to 40 ng/µl for PCR.

### SSR markers design and screening

The *Rps* gene in Wandou 15 was first mapped using the SSR markers described in Soybase (http://soybase.org). Subsequently, new SSR markers were designed according to the sequence between flanking markers Satt543 and Satt615, downloaded from Phytozome (http://www.phytozome.net/soybean), in which the SSR was searched by SSR hunter 1.3 (http://en.bio-soft.net/dna/SSRHunter). The primers were designed using Primer Premier 5.0 (Premier Biosoft International, Palo Alto, CA, USA) with default parameters, based on the simple repeat sequence.

The PCR reaction was performed in a final volume of 15 µl in a TGRADIENT Thermocycler (Biometra, Goettingen, Germany), and comprised 40 ng genomic DNA, 2.0 µl 10×PCR reaction buffer (2.0 mM MgCl_2_) (TIANGEN, Beijing, China), 0.2 mM of each dNTP (TIANGEN), 1.0 U of *Taq* DNA polymerase (TIANGEN) and 0.2 µM of each primer. The PCR amplification consisted of an initial denaturation at 95°C for 5 min; 35 cycles of denaturation at 95°C for 40 s, annealing at temperatures 45–52°C for 40 s and extension at 72°C for 40 s; with a final extension at 72°C for 10 min. PCR products were mixed with 4 µl of 6×loading buffer (0.25% bromophenol blue, 0.25% xylene cyanol FF and 40% sucrose) separated on an 8% polyacrylamide gel.

The polymorphic SSR markers were screened according to the polymorphic DNA fragment between the parents, resistant and susceptible bulks, and further tested in the entire F_2∶3_ mapping population. SSR markers were scored as AA (homozygous for the Wandou 15 allele), BB (homozygous for the Williams allele) or AB (heterozygous) in the 102 F_2∶3_ families.

### Data analysis and genetic linkage map construction

The segregation patterns of phenotypes and SSR markers selected in the mapping population were tested for the goodness-of-fit to Mendelian segregation ratio using Chi-squared (χ^2^) analysis. A genetic linkage map of *Rps10* was constructed with Joinmap 4.0 linkage analysis software [52].

### Candidate gene(s) prediction and sequence analysis

Sequences of candidate gene models within the closely linked SSR markers were obtained from the Phytozome database (http://www.Phytozome.net). And two pairs of primers were designed according to the regions of the 5′-UTR and 3′-UTR of the gene models Glyma17g28950.1 and Glyma17g28970.1, respectively. PCR was performed on genomic DNA using the standard protocol with *Taq* DNA polymerase (TaKaRa, Dalian, China). The allelic variations of the candidate gene(s) in these two cultivars were compared. Sequences alignment was performed with BioEdit software [53]. Gene prediction was performed using the program GENESCAN (http://genes.mit.edu/GENSCAN.html) and verified by Gene Finder (http://rulai.cshl.org/tools/genefinder/) and GenomeScan (http://genes.mit.edu/genomescan.html). InterProScan (http://www.ebi.ac.uk/InterProScan) was used for protein annotation.

## Supporting Information

Figure S1 **Alignment of sequences corresponding to the locus in Wandou 15, Williams amplified using the markers Sattwd15-28 and Sattwd15-32, and the referred region in Glyma17g28950.1 and Glyma17g28970.1.** (A) Alignment of the sequences in Wandou 15, Williams amplified using the marker Sattwd15-28, and the referred region in Glyma17g28950.1 (B) Alignment of the sequences in Wandou 15, Williams amplified using the marker Sattwd15-32, and the referred region in Glyma17g28970.1 (EPS).
(TIF)

Figure S2 **Structures of the gene** ***rpswm-1*** **and** ***rpswm-2*** **, the allelic of** ***Rps10-1*** **and** ***Rps10-2*** **in susceptible cultivar Williams.** (A) Predicated structure of the gene *rpswm-1*. (B) Predicated structure of the gene *rpswm-2*. Filled rectangular arrows with orientations (from 5′ to 3′) indicate the predicated genes. Filled rectangles indicate the exons (EPS).
(TIF)

Table S1 **Annotation of genes between markers Sattwd15-24/25 and Sattwd15-47 with the physical location from 30,796,875 to 31,108.028 on chromosome 17 (DOC).**
(DOC)
