# Supplementary figures and images for: Identification and Candidate Gene Analysis of a Novel Phytophthora Resistance Gene Rps10 in a Chinese Soybean Cultivar
Source: PLoS One. 2013 Jul 25;8(7):e69799. doi: 10.1371/journal.pone.0069799 (PMC3723638; doi:10.1371/journal.pone.0069799)

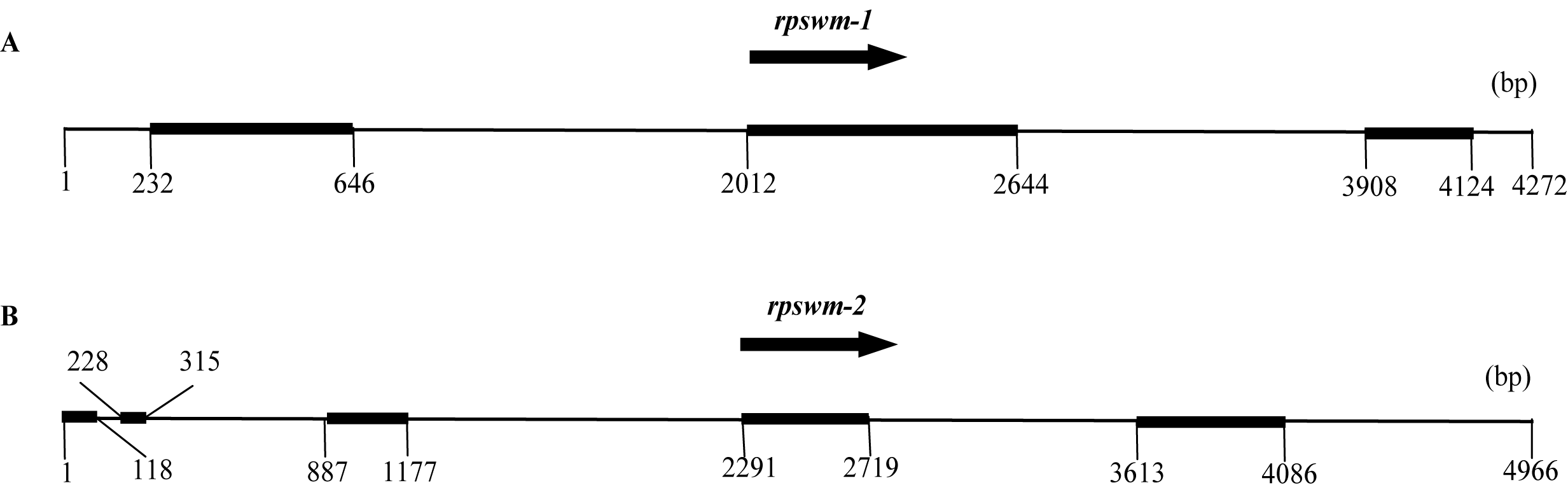

Supplement: Figure S2 — Structures of the gene rpswm-1 and rpswm-2 , the allelic of Rps10-1 and Rps10-2 in susceptible cultivar Williams. (A) Predicated structure of the gene rpswm-1. (B) Predicated structure of the gene rpswm-2. Filled rectangular arrows with orientations (from 5′ to 3′) indicate the predicated genes. Filled rectangles indicate the exons (EPS). (TIF) [file pone.0069799.s002.tif]
