# Supplementary material for: Identification and Candidate Gene Analysis of a Novel Phytophthora Resistance Gene Rps10 in a Chinese Soybean Cultivar
Source: PLoS One. 2013 Jul 25;8(7):e69799. doi: 10.1371/journal.pone.0069799 (PMC3723638; doi:10.1371/journal.pone.0069799)
Supplement: Table S1 — Annotation of genes between markers Sattwd15-24/25 and Sattwd15-47 with the physical location from 30,796,875 to 31,108.028 on chromosome 17 (DOC). (DOC) [file pone.0069799.s003.doc]

Table S1

| No | Gene name | Primer | Position | Gene annotation |
| --- | --- | --- | --- | --- |
| 1 | Glyma17g28740.1 |  | 30839173 - 30848637 | There are no functional annotations for this locus |
| 2 | Glyma17g28950.1 | Sattwd15-28 | 30963677 - 30967974 | Serine-Threonine protein kinase, plant-type |
| 3 | Glyma17g28970.1 | Sattwd15-32 | 30982382 - 30987945 | Serine-Threonine protein kinase, plant-type |
| 4 | Glyma17g28980.1 |  | 30990546 - 30991718 | There are no functional annotations for this locus |
| 5 | Glyma17g29030.1 |  | 31044571 - 31051483 | Prokaryotic DNA topoisomerase |
| 6 | Glyma17g29040.1 |  | 31069990 - 31071879 | There are no functional annotations for this locus |
| 7 | Glyma17g29050.1 |  | 31073708 - 31074317 | There are no functional annotations for this locus |
| 8 | Glyma17g29060.1 |  | 31076408 - 31080054 | Glycosyl transferase family 2 |
